# A recombinant single chain antibody interleukin-2 fusion protein.

**DOI:** 10.1038/bjc.1993.57

**Published:** 1993-02

**Authors:** P. Savage, A. So, R. A. Spooner, A. A. Epenetos

**Affiliations:** Department of Clinical Oncology, Royal Postgraduate Medical School, Hammersmith Hospital, London, UK.

## Abstract

**Images:**


Br. J. Cancer (1993), 67, 304–310 © Macmillan Press Ltd., 1993

### A recombinant single chain antibody interleukin-2 fusion protein

P. Savage[1,2], A. So[2], R.A. Spooner[1] & A.A. Epenetos[1]

*ICRF Monoclonal Targeting Group, Department of Clinical Oncology, Royal Postgraduate Medical School, Hammersmith Hospital, London W12 OHS; [2]Department of Rheumatology, Royal Postgraduate Medical School, Hammersmith Hospital, London W12 OH, UK.*

**Summary** Recombinant interleukin-2 (rIL-2) therapy has been shown to be of value in the treatment of some cases of melanoma and renal cell carinoma. However its use can be limited by severe systemic toxicity. Targeting rIL-2 to the tumour should improve the anti-tumour immune response and decrease the systemic toxicity. With this aim we have employed recombinant DNA techniques to construct a single chain antibody interleukin-2 fusion protein (SCA-IL-2).

The protein used in this model system comprises the variable domains of the anti-lysozyme antibody D1.3 fused to human IL-2. It has been expressed by secretion from *Escherichia coli* and the purified product possesses antigen binding specificity and retains the immunostimulatory activities of rIL-2.

This approach can be taken to generate SCA-IL-2 proteins that bind to appropriate cellular antigens. *In vivo* administration of a tumour binding SCA-IL-2 should result in a localised high concentration of IL-2 in tumour tissues, maximising the anti-tumour immune response, whilst keeping systemic side effects to a minimum.

Interleukin-2 (IL-2) is a 15 kDa cytokine produced by T helper cells that stimulates cytotoxic T lymphocytes and NK cells (Gillis *et al.*, 1978). Bacterially produced recombinant IL-2 has been used clinically in the treatment of melanoma and renal cell carcinoma to stimulate cancer patients' immune systems (Rosenberg *et al.*, 1989). Recent pre-clinical studies indicate that achieving a prolonged high dose of IL-2 in the tumour can result in the induction of a long lasting anti-tumour response leading to the rejection of an otherwise lethal tumour (Fearon *et al.*, 1990). However the *in vivo* efficacy is limited by difficulties in maintaining prolonged high doses in the tumour and by the severe systemic toxicity associated with high dose IL-2 therapy (Rosenberg *et al.*, 1989).

To achieve a selective and prolonged concentration of IL-2 in the tumour it is an attractive idea to target it there via an antibody delivery system. IL-2 has been successfully incorporated into a number of fusion proteins. Fusion proteins consisting of IL-2 linked to bacterial toxins have been produced in bacteria and have been demonstrated to be toxic to IL-2 receptor bearing cells (Lorderboum-Galski *et al.*, 1988; Williams *et al.*, 1987). With the aim of concentrating IL-2 activity in the tumour, two different antibody-IL-2 fusion proteins have already been described. A Fab′-IL-2 fusion protein, whilst only partially retaining IL-2 activity, has been shown to increase the T-cell mediated killing of antigen-bearing tumour cells *in vitro* (Fell *et al.*, 1991). A larger IgG-IL-2 fusion protein appears to retain full IL-2 activity and is likewise able to increase effector cell mediated killing *in vitro* (Gillies *et al.*, 1992). There are potential problems with these molecules in that they are produced in expensive mammalian expression systems and that their large size may result in poor tumour penetrance and prolonged blood residues *in vivo*. A smaller antibody-IL-2 fusion protein based on a bacterially produced antibody fragment may give economic and therapeutic advantages.

As a delivery system the smaller single chain antibody (SCA), comprising linked variable heavy ($V_H$) and variable light ($V_L$) chain antibody domains shows great promise (Huston *et al.*, 1988). Where tested SCAs demonstrate good tissue penetration (Yokota *et al.*, 1992), rapid renal clearance of non-localised protein and potentially low immunogenicity (Colcher *et al.*, 1990). Recent advances with *in vitro* selection should allow the rapid and economic production of SCA of any required specificity (Clackson *et al.*, 1991; Marks *et al.*, 1991). In this preliminary investigation the feasability of producing a functional SCA-IL-2 was tested using the anti-lysozyme SCA D1.3. The advantages of using this antibody are that it is well characterised, it is secreted well and it is easy to detect by Elisa and to affinity purify. As we employed a SCA unsuitable for cytotoxicity assays we are not able to add to the already strong evidence for the benefits of concentrating IL-2 in tumours. However we are able to demonstrate for the first time that this fusion protein, in addition to retaining antigen binding ability, possesses the immunostimulatory actions of IL-2 when tested with lymphocytes bearing the high affinity IL-2 receptor. Furthermore the SCA-IL-2 fusion molecule described here retains the ability to stimulate cells expressing low affinity IL-2 receptors as measured by its proliferative effects on human peripheral blood lymphocytes.

### Materials and methods

### *Plasmid assembly*

Single colonies of *E.coli* containing plasmid pSV-HIL2-0 (Gift of Professor W. Friers, University of Ghent) were suspended in 500 µl of water, boiled for 5 min and cleared by centrifugation in a microfuge. Aliquots (10 µl) of the supernatant were subjected to polymerase chain reaction (PCR) amplification according to the manufacturers instructions (Perkin Elmer Cetus, Norwalk, USA) in the presence of 25 pmol each of oligonucleotide primers IL-2/7 and IL-2 3′. The reaction underwent 30 cycles of denaturation (94°C, 1 min), annealing (50°C, 1 min) and extension (72°C, 1 min). Oligonucleotide primer IL-2/7, (5′-ACCAAGCTCGAGATC-AAACGGGAACAAAAACTCCCTACTTCAAGTTCT-3′) direct incorporation of an *Xho* I site and the seven carboxy-terminal amino acids of the $V_L$ chain domain of the D1.3 SCA plasmid (pSWsFVD1.3myc, McCafferty *et al.*, 1990; Gift of Dr E.S. Ward, LMB, Cambridge) fused to sequence encoding amino acids 2–7 of human IL-2. Primer IL-2 3′, (5′-TTCTCGAATTCGAGCTGGATCCTTATTAAGTCAG-TGTTGAGATGAT-3′), directs the incorporation of an *Eco*RI site downstream of the termination codon of human IL-2. The 440 bp amplified product was isolated from a 1.5% agarose gel, digested with *Xho* I and *Eco*RI and ligated between the *Xho* I and *Eco*RI sites of plasmid pSWsFVD 1.3myc to generate plasmid pSCA-IL-2/7. This plasmid bears a chimeric gene encoding a single chain antibody-IL-2 fusion protein (SCA-IL-2) under *lac* transcriptional control. Figure 1 shows plasmids used, the nucleotide sequence and the deduced amino acid sequence at the fusion junction.

Correspondence: P. Savage, ICRF Department of Clinical Oncology, Hammersmith Hospital, Du Cane Road, London W12 OHS, UK.
Received 13 May 1992; and in revised form 15 September 1992.

**Figure 1** Construction of plasmid pCA-IL-2/7. Upper: amplification and modification of the IL-2 gene; middle: replacement of the *c-myc*-derived portion with the modified IL-2 gene (not to scale); and lower: nucleotide and derived amino-acid sequences of the non-transcribed strand at the junction between the segments encoding antibody domains and the IL-2 gene. Plasmids pSCA-IL-2/7 and pSWsFVD1.3myc are pUC19 derivatives. Only the sections between the *Hind*III and *Eco*RI sites of the cloning region are shown.

### *Expression and partial purification of SCA-IL-2 protein*

Cultures (500 ml) of *E.coli* K12 KS476 (Stauch *et al.*, 1989; Gift of Professor J. Beckwith, Harvard) transformed with plasmid pSCA-IL-2/7 were grown overnight at 37°C in 2 × TY broth supplemented with appropriate antibodies and, to ensure growth in repressing conditions, 1% glucose. Cells were harvested, washed twice in sterile standard phosphate buffered saline (PBS pH 7.6) at 27°C, suspended in fresh growth media containing appropriate antibiotics, supplemented with 0.1 mM isopropyl-b-D-thiogalactopyranoside (IPTG), and shaken for 16 h at 25°C to permit accumulation of the fusion protein. After expression, cells were harvested, the bacterial growth supernatant was filtered (0.22 mm filter) and applied at room temperature to a lysozyme-Sepharose column. After washing with PBS, bound protein was eluted as described previously (Ward *et al.*, 1989). Prior to use the fusion protein was dialysed exhaustively against PBS and stored at − 20°C.

### *ELISAs*

For serological detection of SCA-IL-2 fusions proteins, enzyme linked immunosorbent assays (ELISAs) were employed. Flat bottomed Dynatech Immulon 96 well plates were coated overnight at 25°C with hen egg lysozyme (300 $\mu g\ ml^{-1}$), goat polyclonal anti-IL-2 antibody (50 $\mu g\ ml^{-1}$, British Biotechnology Oxford) or other proteins (BSA, KLH, reconstituted milk powder, or insulin at appropriate concentrations) applied in 50 mM bicarbonate buffer pH 9.6. Unoccupied sites were blocked with a 1% solution of milk powder in PBS for 30 min at 25°C. Bacterial supernatants, affinity purified material or recombinant IL-2 (rIL-2, Boehringer Mannheim, Germany) were diluted in PBS/1% milk powder and incubated (30 min, 25°C) in appropriate wells. After three washes in PBS, bound protein was detected with either polyclonal anti IL-2 or DMS1 (Smith *et al.*, 1983), a murine monoclonal antibody that recognises the receptor binding site of human IL-2 (Gift of Professor K.A. Smith, Dartmouth Medical School, USA). After a further three washes, bound antibody was detected with a species specific HRP conjugated antibody (DAKO, Copenhagen, Denmark). ABTS was added to generate a colour change that was monitored at 405 nm.

### *Western blots*

Affinity purified protein samples were electrophoresed through 15% 30:1 acrylamide: bis-acrylamide gels essentially as described in Laemmli (Laemmli, 1970) and transferred electrophoretically to a nitrocellulose membrane (Towbin *et al.*, 1979). The membrane was blocked (30 min, 25°C) in a 1% solution of milk powder in Tris-buffered saline/0.0025% Tween 20 (TBST). Proteins were detected by incubation (1 h, 25°C) with either rabbit anti-SCA serum (Gift of E.S. Ward) or mouse monoclonal DMS1. After five washes in TBST, bound first step antibody was detected with anti-rabbit or anti-mouse AP conjugated antibody (Amersham, Bucks, UK) and revealed by incubation with a solution of NBT and BCIP (Promega, Madison, USA), according to the manufacturers recommendations.

### *FACS analysis*

CTLL-2 cells (Gillis & Smith, 1977) deprived of IL-2 for 12 h were seeded into Nunc 96 well plates at $10^5$ per well in 200 $\mu l$ volumes. To the cells was added either a 1:15 dilution of fusion protein, approximating to an activity of 100 U IL-2 $ml^{-1}$, an equivalent dilution of native SCA, rIL-2 at 100 U $ml^{-1}$ or a mixture of SCA and rIL-2. For competitive inhibition, cells were exposed to 5000 U $ml^{-1}$ of rIL-2 or

TNF at 10 μg $ml^{-1}$ for 10 min prior to the addition of SCA-IL-2 as above. After incubation, cell associated SCA epitopes were detected by incubation with rabbit anti-SCA serum and then the bound rabbit anti-SCA antibodies were detected with FITC-conjugated anti-rabbit IgG (Sigma). After fixing with 1% paraformaldehyde, cell surface fluourescence was measured using a Becton Dickinson FACScan. Cells were washed five times between steps with RPMI to remove unbound material, and all incubations were for 30 min at 4°C.

*Bioactivity assays*

CTLL-2 cells (Gillis et al., 1978), which bear the high affinity IL-2 receptor, were maintained in RPMI media supplemented with 10% foetal calf serum (heat inactivated) and 10 U $l^{-1}$ rIL-2. For assay, cells were washed in media and deprived of IL-2 for 4 h, after which they were seeded into 96 well plates at $5 \times 10^3$ per well. Dilutions of fusion protein or rIL-2 were added and the cultures incubated for 18 h at 37°C in a 5% $CO_2$ atmosphere. Then to each well 0.5 μCi of $^3H$-thymidine (Amersham) was added. After a further 4 h incubation cells were harvested onto glass fibre filters, dried and the incorporated radioactivity counted. For inhibition assays, fusion protein or rIL-2 at five times the concentration that produced 50% maximal stimulation of CTLL-2 cells was incubated with dilutions of goat anti-IL-2 antibody (30 min, 37°C) prior to addition to the CTLL-2 cells, for assay as described above.

Peripheral blood lymphocytes were obtained by venepuncture from healthy donors and prepared by differential centrifugation using Lymphoprep (Nycomed, Oslo, Norway). After washing in RPMI media and seeding into tissue culture plates at $10^5$ cells per well, appropriate dilutions of fusion protein or rIL-2 were added. Following incubation (36 h, 37°C, 5% $CO_2$), 0.5 μCi of $^3H$-thymidine was added to each well, and after 4 h further incubation cells were harvested and incorporated radioactivity was measured.

### Results

*Expression and affinity chromatography of SCA-IL-2/7 protein*

SCA-IL-2/7 protein expression was induced by addition of 0.1 mM IPTG to transformed cultures of *E.coli* K12 KS476. Figure 2a shows a Western Blot of material affinity-purified from culture growth medium detected with the anti-IL-2 antibody DMS1; this reveals a single band with an apparent molecular weight of 46 kDa. When probed with anti-SCA serum, a 46 kDa band was still apparent, but a number of degradation products were revealed (Figure 2b). As DMS1 binds the carboxyl end of IL-2 and does not recognise any of the degradation products we assume that proteolysis has removed at least the terminal carboxyl section of the fusion protein in these degradation products.

The ability of SCA-IL-2/7 fusion protein to bind lysozyme is demonstrated in Figure 3, where affinity purified material was allowed to bind immobilised lysozyme and was detected with polyclonal anti-IL-2. Furthermore, against the panel of immobilised protein antigens tested in Figure 4 there is no evidence of the non-specific stickiness sometimes associated with antibody fragments.

In Elisas (Figure 5) in which SCA-IL-2/7 or rIL-2 are immobilised on the polyclonal anti-IL-2, the dose response curves generated with DMS1 are similar for both the fusion protein and rIL-2. An estimate of the serological activity of the IL-2 activity of the SCA-IL-2 sample used in these experiments is 1200–1500 U $ml^{-1}$.

*FACS analysis*

FACS analysis was used to determine if the SCA-IL-2 protein is able to interact with the IL-2 receptors of lymphoid cells as an intact protein rather than a degraded form consisting of its two parent molecules. Cell surface bound SCA epitopes can only be demonstrated in the presence of SCA-IL-2 fusion protein (Figure 6). The fusion protein gives a

**Figure 2** Western blot analysis of fusion protein. **a**, Proteins recognised by antibody DMS-1. Lane A; molecular weight markers; lane B, rIL-2, and lane C, affinity-purified SCA-IL-2/7. **b**, Proteins recognised by anti-SCA. Lane D molecular weight markers; lane E, native SCA, and lane F, affinity-purified SCA-IL-2/7.

**Figure 3** Detection of SCA-IL-2/7 protein by ELISA. Bacterial culture supernatants of cells transformed with pSWsFVD1.3myc encoding anti-lysozyme single chain antibody (SCA), pSCA-IL-2/7 encoding Single chain antibody IL-2 fusion protein and pUC19 were diluted 1:2 and applied to microtitre plates previously coated with hen egg lysozyme. Bound IL-2 epitopes were detected using anti-IL-2.

**Figure 4** Binding of pSCA-IL-2/7 culture supernatant to a panel of protein antigens. Bound proteins were recognised with goat anti-IL-2 sera.

significant rise in fluorescence compared with the negative control, whilst free SCA either alone or with free rIL-2 give no increase on the background value. The specificity of the fusion protein interaction with the cells is demonstrated by the reduction of fluorescence almost to background levels in the presence of excess free rIL-2/ This competitive inhibition of SCA-IL-2 binding by free rIL-2 demonstrates that the fusion protein binds to the cells through specific receptor-ligand interactions. Competition with an excess of a non-specific protein (TNF) had no effect on the level of SCA-IL-2 binding (Data not shown).

Results from sequential Elisas and western blots also indicate that the fusion protein is stable under the conditions of the biological assays described (Data not shown).

**Figure 5** Quantitation of the full length IL-2 epitopes as detected by mAb DMS1 in rIL-2 and SCA-IL-2/7 immobilised on polyclonal anti-IL-2.

**Figure 6** Facs analysis of interactions of SCA-IL-2 fusion protein and CTLL-2 cells, by detection of cell surface bound SCA epitopes.

### *Stimulation of CTLL-2 cells and peripheral blood lymphocytes (PBLs) by SCA-IL-2/7 protein*

In the conditions employed, half-maximal stimulation of IL-2 dependent CTLL-2 cells occurred at a concentration of approximately 0.4 U $ml^{-1}$ rIL-2 (Figure 7). SCA-IL-2/7 protein gave a similar maximal stimulation and dose response curve. The fusion protein sample achieved a similar half-maximal stimulation at a 5120 fold dilution, giving activity of the affinity purified material of approximately 1500 U $ml^{-1}$ rIL-2 against cells bearing the high affinity IL-2 receptor. This figure is in close agreement with the estimate derived from the serological assay. Goat anti-rIL-2 sera inhibited the proliferative effects of both rIL-2 and SCA-IL-2/7 protein in a similar manner, giving complete inhibition at 25 mg $ml^{-1}$ (Figure 8).

The effects of rIL-2 and SCA-IL-2/7 protein on peripheral blood lymphocytes, that bear the low and intermediate affinity forms of the IL-2 receptor, are shown in Figure 9. The dose response curve illustrates that SCA-IL-2 protein acted in a similar manner to IL-2 and the activity of the affinity purified material corresponds to approximately 1600 U $ml^{-1}$ rIL-2, again in close agreement with previous estimates. Native D1.3 SCA prepared by identical methods had no proliferative action in either assay (Data not shown).

### Discussion

In this study we report the construction, expression and characterisation of a novel fusion protein, SCA-IL-2/7, that retains both the antigen binding characteristics of the parent single chain antibody and the immuno-stimulatory actions of IL-2. The genetic construct encodes the *pel*B leader sequence (Lei *et al.*, 1987) that directs the expressed protein to the bacterial periplasm, where the oxidising environment should permit the formation of intramolecular disulphide bonds. Whilst the majority of material produced by the bacteria was degraded by proteases to give a product similar to the native SCA, a significant quantity of functional material was obtained from the culture supernatant and partially purified by affinity chromatography. The yield from the current system is very low, but it is apparent from experience with similar Fv fragments that yields can be optimised to produce a many fold improvement (Better *et al.*, 1990).

We have demonstrated that the fusion protein retains antigen binding ability and that it is able to stimulate lymphoid cells bearing IL-2 receptors. When tested by serological and biological assays the fusion protein and IL-2 gave similar dose response curves: incorporation of IL-2 into this fusion protein appears not to reduce its activity as it has in a previously described Fab'IL-2 fusion (Fell *et al.*, 1991). It may be that the smaller SCA-IL-2 protein permits efficient receptor complex internalisation. This fusion protein interacts with and stimulates cells bearing either high or low affinity IL-2 receptors. The dual specificity may be allowed by the relatively long linker between the antibody domains and the IL-2 moiety allowing the amino-terminal end of IL-2 freedom to interact with the low affinity receptor. Work with diptheria toxin-IL-2 fusion proteins has demonstrated the importance of this mobility in allowing this interaction to occur effectively (Williams *et al.*, 1987; Kiyokawa *et al.*, 1991).

We have demonstrated that the SCA-IL-2 protein is stable under the conditions of the purification and assay and the FACS results demonstrate that it is intact when it interacts with the IL-2 receptor on the CTLL-2 cell's surface. In addition, work in progress demonstrates that the fusion protein, like IL-2, is able to cause an increase in vascular permeability (Savage *et al.*, 1993). Although IL-2 induced permeability on a systemic scale can lead to toxicity (Lotze *et al.*, 1986), as a localised event it can enhance the passage of itself and other therapeutic macromolecules into the tumour and ease access for effector cells (LeBerthon *et al.*, 1991; Hennigan *et al.*, 1991).

**Figure 8** Inhibition of stimulatory effects of rIL-2 and SCA-IL-2/7 by polyclonal anti-IL-2 sera.

**Figure 7** Stimulatory effects of rIL-2 and SCA-IL-2/7 fusion protein on high affinity IL-2 receptor-bearing CTLL-2 cells. Proliferation is measured by incorporation of $^{3}H$-thymidine.

**Figure 9** Stimulatory effects of rIL-2 and SCA-IL-2/7 fusion protein on human PBLs, measured by incorporation of $^3$H-thymidine.

Our aim is to target, via antibody variable domains, IL-2 activity to cells of the immune system in the area of the tumour. Accumulation of IL-2 around any inappropriately targeted normal cells should result in little cytolytic action, since effector cells do not interact significantly with normal cells. This approach should therefore much reduce the requirement for highly selective tumour associated antigens. This contrasts with the potentially detrimental effects of radionucleide- or toxin-conjugated antibodies binding normal cells. Whilst the specificity of the current fusion protein is only suitable for preliminary *in vitro* experiments, recent advances in *in vitro* selection of antibody variable regions should allow the rapid generation of SCA directed against appropriate cellular targets.

Encouraging pre-clinical results with rIL-2 have only been partially supported by clinical experience. Poor clinical responses are in part owing to failure to achieve long lasting therapeutic concentrations in target tissues and also the systemic toxicity associated with large doses. Targeting of IL-2 by an antibody-derived fusion protein should allow concentration and prolonged action of rIL-2 within the area of the tumour whilst minimising systemic toxicity. Since the SCA-IL-2 described here may interact the low affinity IL-2 receptors on resting PBLs it is unlikely that it will localise effectively following intra-venous administration. However regional or direct intra-tumoural administration may result in accumulation, prolonged residence and an increased anti-tumour immune response in the tumour. For IV administration and localisation it will be preferable to have a form that interacts only with the high affinity IL-2 receptor, as expressed on NK cells and activated T-cells. Work is in progress to determine if shortening the linker in the SCA-IL-2 construct will produce a protein that will selectively stimulate cells bearing the high affinity receptor. SCA-IL-2 fusion proteins may provide an effective method of targeting therapeutic doses of rIL-2 to tumours or other targeted cells whilst significantly reducing systemic toxicity.

### References

BETTER, M., WEICKMANN, J. & LIN, Y.-L. (1990). Production and scale up of chimeric Fab fragments from bacteria. *ICSU Short report* 10, 105. IRL press: Oxford University Press.

CLACKSON, T., HOOGENBOOM, H.R., GRIFFITHS, A.D. & WINTER, G. (1991). Making antibody fragments using phage display libraries. *Nature*, **352**, 624-628.

COLCHER, D., BIRD, R., ROSELLI, M., HARDMAN, K.D., JOHNSON, S., POPE, S., DODD, S., PANTOLIANO, M.W., MILENIC, D.E. & SCHLOM, J. (1990). In vivo tumour targeting of a recombinant single-chain antigen-binding protein. *JNCI*, **82**, 1191-1197.

FEARON, E.R., PARDOLL, D.M., ITAYA, T., GOLUMBEK, P., LEVITSKY, H.I., SIMONS, J.W., KARASUYAMA, H., VOGELSTEIN, B. & FROST, P. (1990). Interleukin-2 production by tumour cells bypasses T helper function in the generation of an antitumour response. *Cell*, **60**, 397-403.

FELL, H.P., GAYLE, M.A., GROSMAIRE, L. & LEDBETTER, J.A. (1991). Genetic construction and characterization of a fusion protein consisting of a chimeric F(ab') with specificity for carcinomas and human IL-2. *J. Immunol.*, **146**, 2446-2452.

GILLIES, S.D., REILLY, E.D., LO, K.M. & REISFEL, R.A. (1992). Antibody-targeted interleukin 2 stimulates T-cell killing of autologous tumor cells. *Proc. Natl Acad. Sci. USA*, **89**, 1428-1432.

GILLIS, S., FERM, M.M., OU, W. & SMITH, K.A. (1978). T cell growth factor: parameters of production and a quantative microassay for activity. *J. Immunol.*, **120**, 2027-2032.

GILLIS, S. & SMITH, K.A. (1977). Long term culture of tumour-specific cytotoxic T cells. *Nature*, **268**, 154-156.

HENNIGAN, T.W., BEGENT, R.H.J. & ALLEN-MERSH, T.G. (1991). Histamine, leukotriene C4 and interleukin-2 increase antibody uptake into a human carcinoma xenograft model. *Br. J. Cancer.*, **64**, 872-874.

HUSTON, J.S., LEVINSON, D., MUDGETT-HUNTER, M., TAI, M.-S., NOVOTNY, J., MARGOLIES, M.N., RIDGE, R.J., BRUCCOLERI, R.E., HABER, E., CREA, R. & OPPERMAN, H. (1988). Protein engineering of antibody binding sites: recovery of specific activity in an anti-digoxin single-chain Fv analogue produced in Escherichia coli. *Proc. Natl. Acad. Sci. USA*, **85**, 5879-5883.

KIYOKAWA, T., WILLIAMS, D.P., SNIDER, C.E., STROM, T.B. & MURPHYS, J.R. (1991). Protein engineering of diptheria-toxin-related interleukin-2 fusion toxins to increase cytotoxic potency for high affinity IL-2-receptor bearing target cells. *Prot. Engin.*, **4**, 463-468.

LAEMMLI, U.K. (1970). Cleavage of structural proteins during the assembly of the head of bacteriophage T4. *Nature*, **227**, 680-685.

LANDOLF, N.F. (1991). A chimeric IL-2/Ig molecule possesses the functional activity of both proteins. *J. Immunol.*, **146**, 915-919.

LEBERTHON, B., KHAWLI, L.A., ALAUDDIN, M., MILLER, G.K., CHARAK, B.S., MASUMDER, A. & EPSTEIN, A.L. (1991). Enhanced tumour uptake of macromolecules induced by a novel vasoactive interleukin-2 immunoconjugate. *Cancer Res.*, **51**, 2694-2698.

LEI, S.P., LIN, H.C., WANG, S.S., CALLAWAY, J. & WILCOX, G. (1987). Characterization of the *Erwinia carotovora pel*B gene and its product pectate lyase. *J. Bact.*, **169**, 4379-4383.

LORDERBOUM-GALSKI, H., FITZGERALD, D., CHAUDARY, V., ALDHAYA, S. & PASTAN, I. (1988). Cytotoxic activity of an interleukin-2-Pseudomonas exotoxin chimeric protein produced in *Escherichia coli*. *Proc. Natl Acad. Sci. USA*, **85**, 1922–1926.

LOTZE, M.T., CHANG, A.G., SEIPP, C.A., SIMPSON, C., VETTO, J.T. & ROSENBERG, S.A. (1986). High dose recombinant interleukin 2 in the treatment of patients with disseminated cancer. *J. Am. Assoc.*, **256**, 3117–3124.

MARKS, J.D., HOOGENBOOM, H.R., BONNERT, T.P., MCCAFFERTY, J., GRIFFITH, D. & WINTER, G. (1991). By-passing immunization. Human antibodies from V-gene libraries displayed on phage. *J. Mol. Biol.*, **222**, 581–597.

MCCAFFERTY, J., GRIFFITHS, A.D., WINTER, G. & CHISWELL, D.J. (1990). Phage antibodies: filamentous phage displaying antibody variable domains. *Nature*, **348**, 552–554.

ROSENBERG, S.A., LOTZE, M.T., YANG, J.A., AEBERSOLD, P.M., LINEHAN, W.M., SEIPP, C.A. & WHITE, D.E. (1989). Experience with the use of high dose interleukin-2 in the treatment of 652 cancer patients. *Ann. Surg.*, **210**, 474–485.

SAVAGE, P.M., BEYNON, H.C., SO, A., WALPORT, M.J. & EPENETOS, A.A. (1993). The single chain antibody interleukin-2 fusion protein mimics the effects of rIL-2 on endothelial cell permeability (submitted).

SMITH, K.A., FAVATA, M.F. & OROSZLAN, S. (1983). Production and characterization of monoclonal antibodies to human interleukin-2: strategy and tactics. *J. Immunol.*, **131**, 1808–1815.

STAUCH, K.L., JOHNSON, K. & BECKWITH, J. (1989). Characterization of degP: a gene required for proteolysis in the cell envelope and essential for growth of *Escherichia coli* at high temperature. *J. Bact.*, **171**, 2689–2696.

TOWBIN, H., STAEHELIN, T. & GORDON, J. (1979). Electrophoretic transfer of proteins from polyacrylamide gels to nitrocellulose sheets: procedure and some applications. *Proc. Natl Acad. Sci. USA*, **76**, 4350–4354.

WARD, E.S., GUSSOW, D., GRIFFITHS, A.D., JONES, P.T. & WINTER, G. (1989). Binding activities of a repertoire of single immunoglobulin variable domains secreted from *Escherichia coli*. *Nature*, **341**, 544–546.

WILLIAMS, D.P., PARKER, K., BACHA, P., BISHAI, W., BOROWSKI, M., GENBAUFFE, F., STROM, T.B. & MURPHY, J.R. (1987). Diptheria toxin receptor binding domain substitution with interleukin-2: genetic construction and properties of a diptheria toxin-related interleukin-2 fusion protein. *Prot. Engin.*, **1**, 493–498.

YOKOTA, T., MILENIC, D.E., WHITLOW, M. & SCHLOM, J. (1992). Rapid tumour penetration of a single-chain Fv and comparison with other immunoglobulin forms. *Cancer Res.*, **52**, 3402–3408.


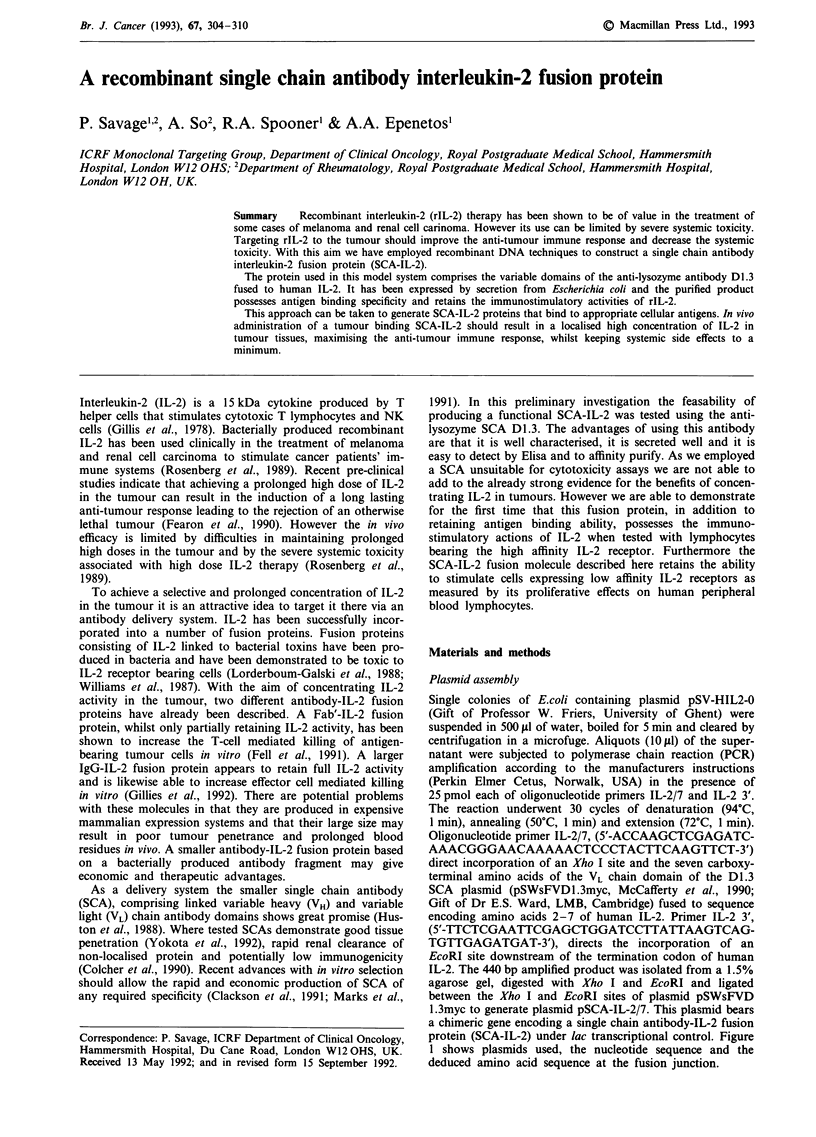

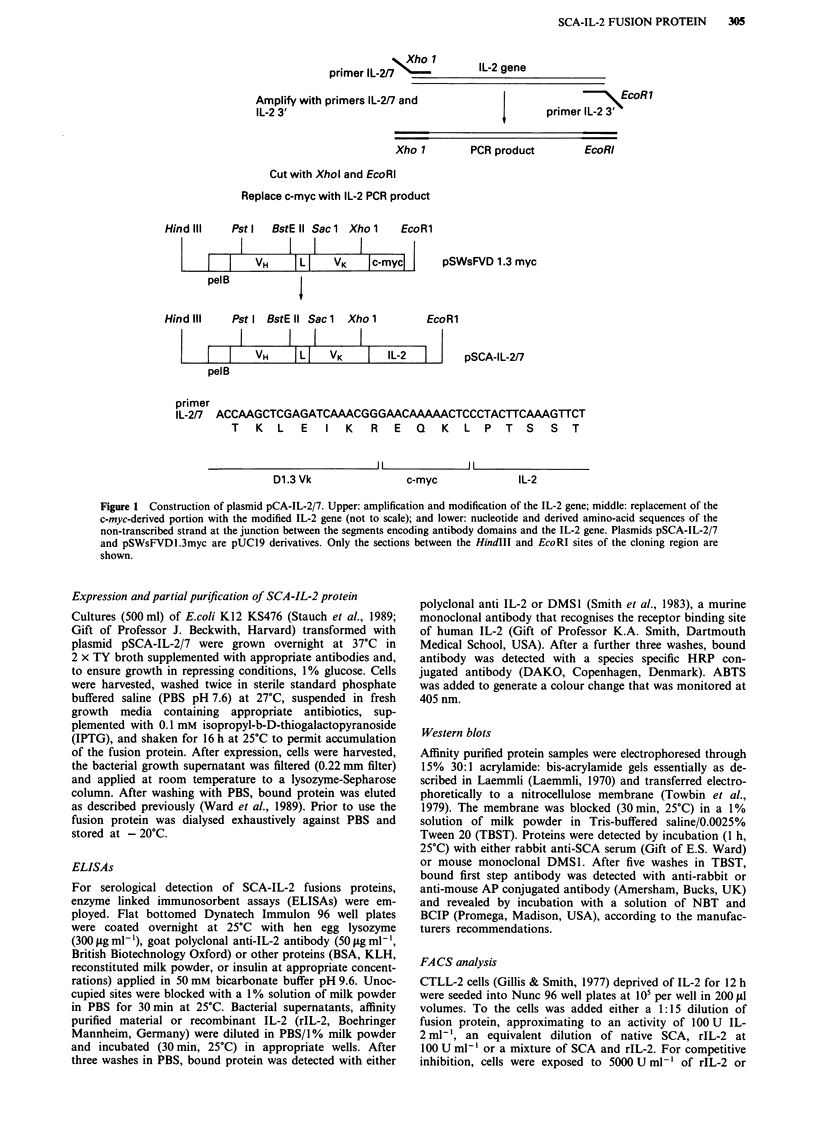

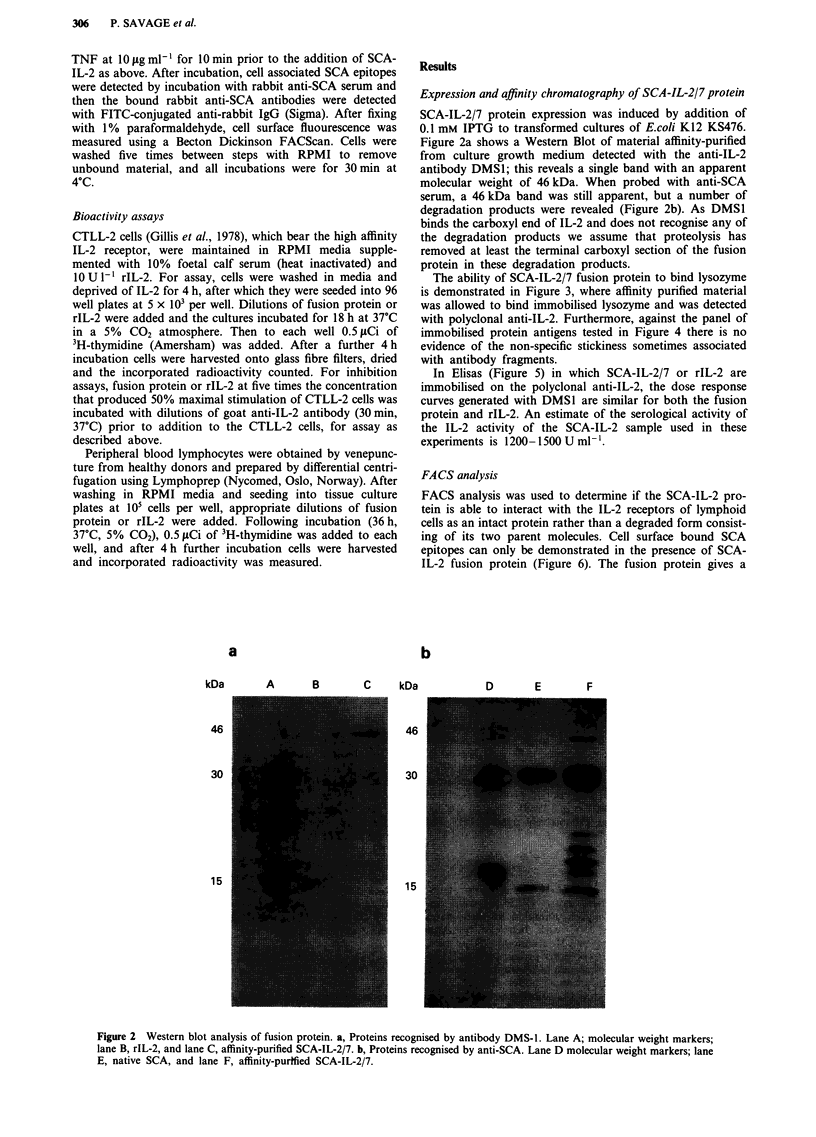

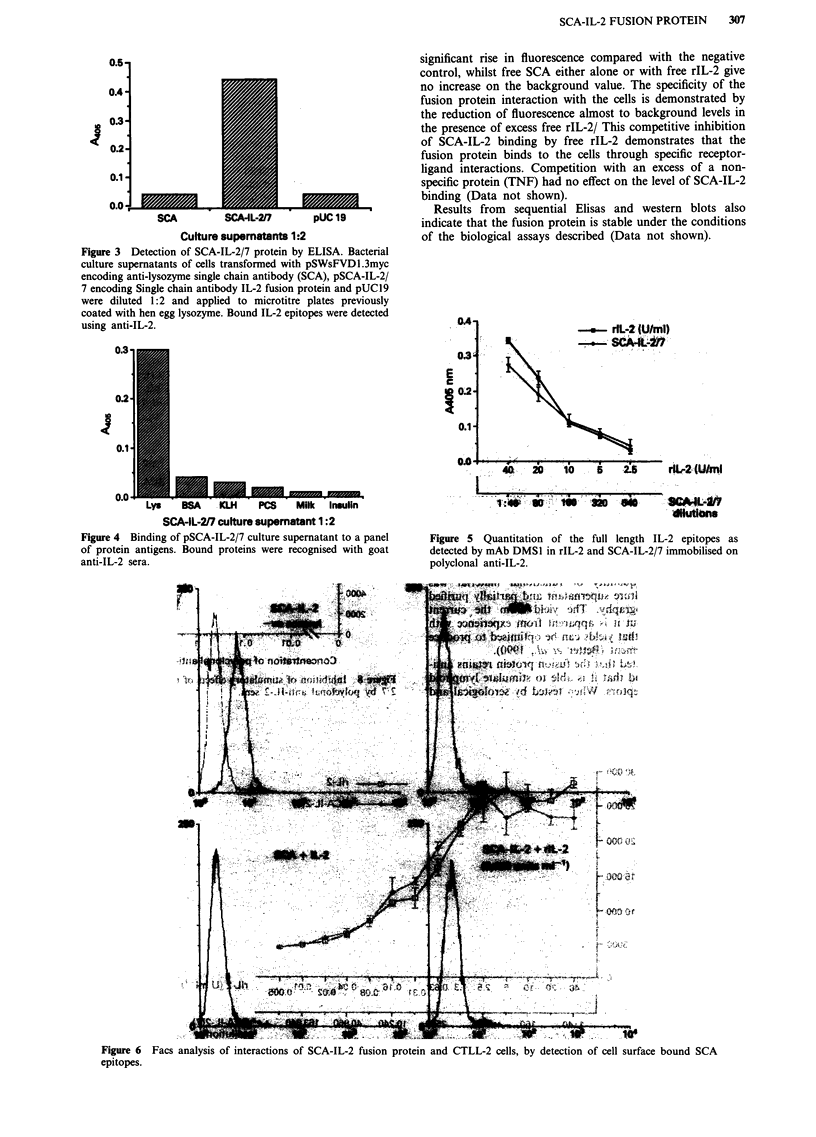

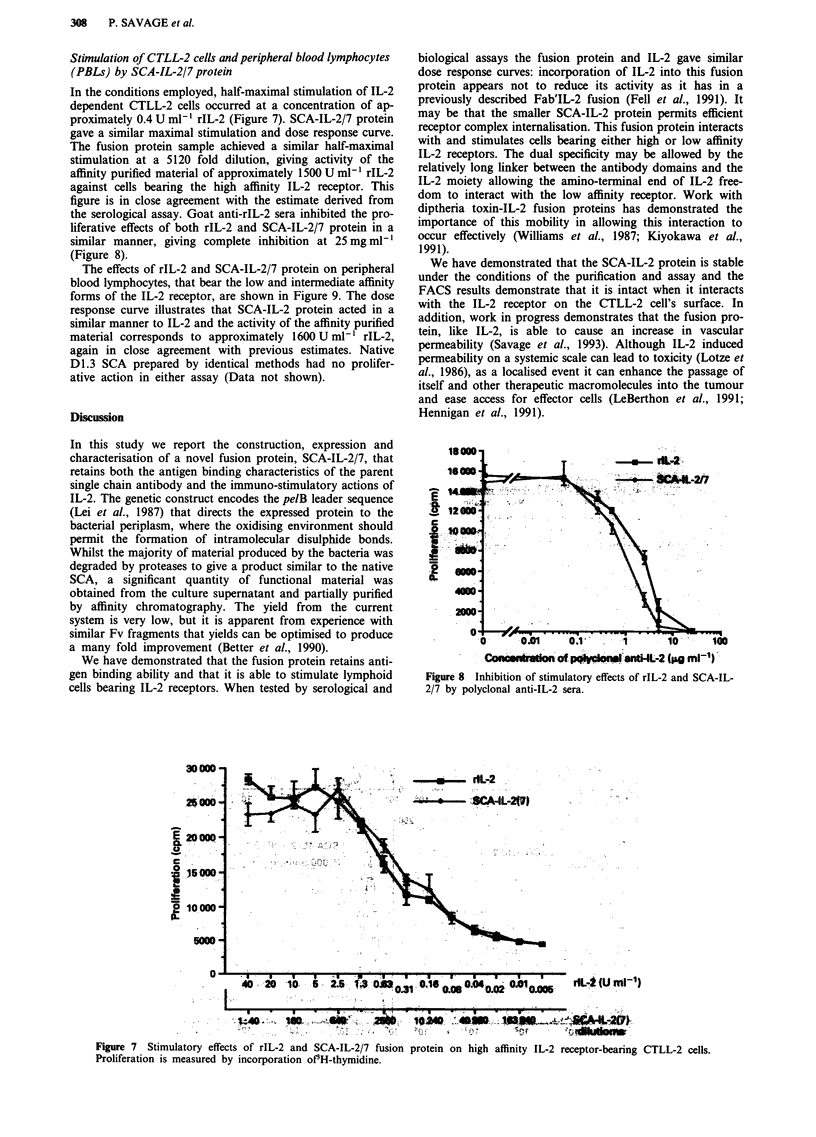

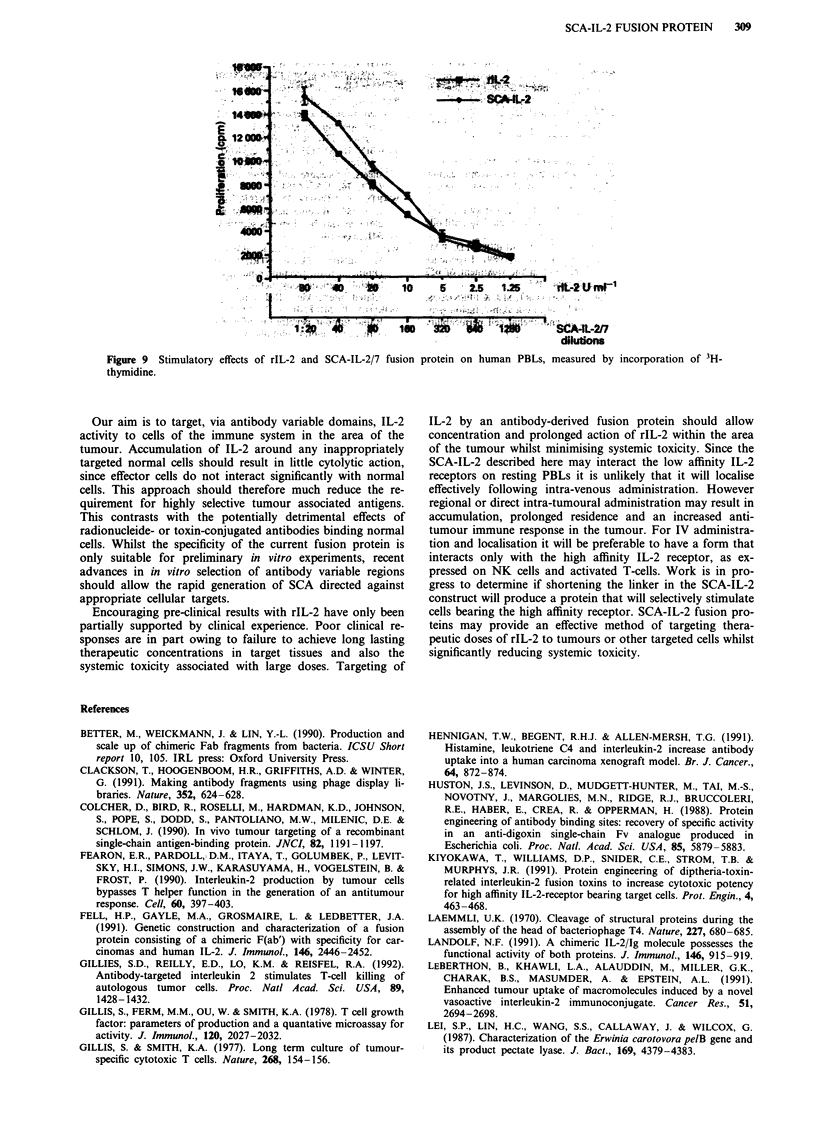

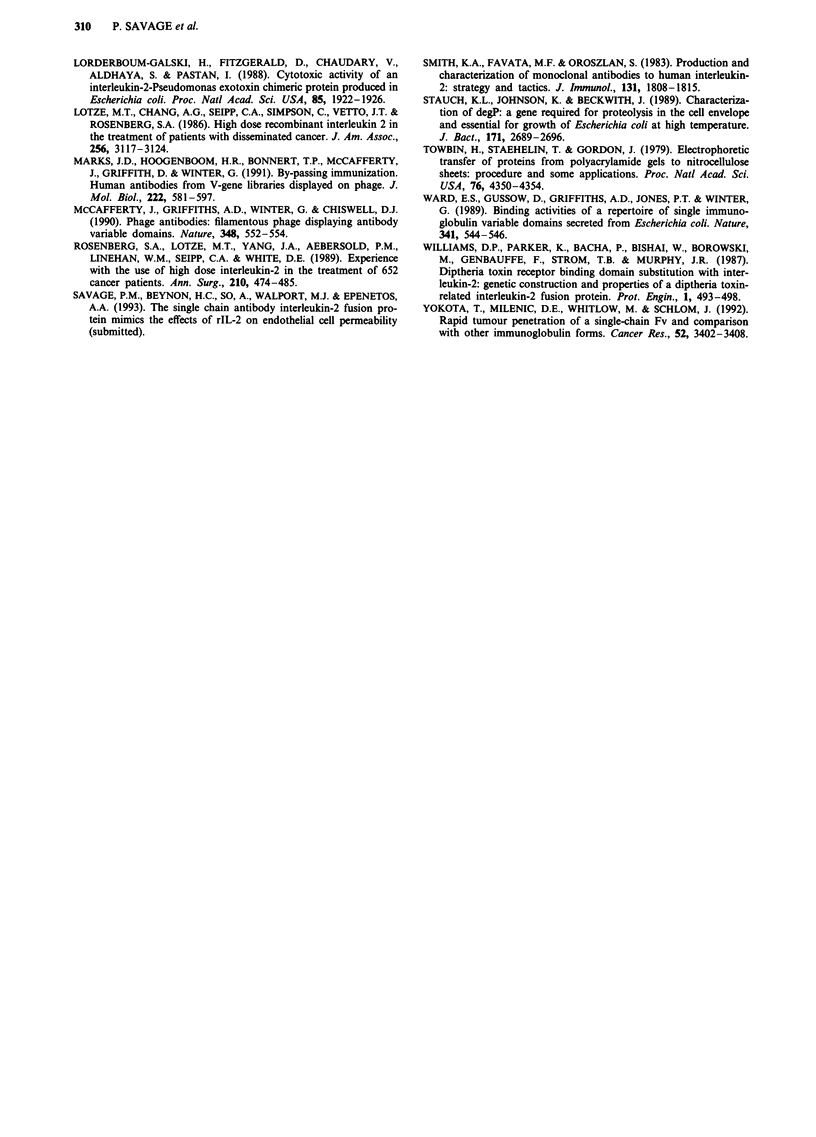


## References

[OCR_00690] Clackson T., Hoogenboom H. R., Griffiths A. D., Winter G. (1991). Making antibody fragments using phage display libraries.. Nature.

[OCR_00695] Colcher D., Bird R., Roselli M., Hardman K. D., Johnson S., Pope S., Dodd S. W., Pantoliano M. W., Milenic D. E., Schlom J. (1990). In vivo tumor targeting of a recombinant single-chain antigen-binding protein.. J Natl Cancer Inst.

[OCR_00703] Fearon E. R., Pardoll D. M., Itaya T., Golumbek P., Levitsky H. I., Simons J. W., Karasuyama H., Vogelstein B., Frost P. (1990). Interleukin-2 production by tumor cells bypasses T helper function in the generation of an antitumor response.. Cell.

[OCR_00708] Fell H. P., Gayle M. A., Grosmaire L., Ledbetter J. A. (1991). Genetic construction and characterization of a fusion protein consisting of a chimeric F(ab') with specificity for carcinomas and human IL-2.. J Immunol.

[OCR_00714] Gillies S. D., Reilly E. B., Lo K. M., Reisfeld R. A. (1992). Antibody-targeted interleukin 2 stimulates T-cell killing of autologous tumor cells.. Proc Natl Acad Sci U S A.

[OCR_00720] Gillis S., Ferm M. M., Ou W., Smith K. A. (1978). T cell growth factor: parameters of production and a quantitative microassay for activity.. J Immunol.

[OCR_00725] Gillis S., Smith K. A. (1977). Long term culture of tumour-specific cytotoxic T cells.. Nature.

[OCR_00729] Hennigan T. W., Begent R. H., Allen-Mersh T. G. (1991). Histamine, leukotriene C4 and interleukin-2 increase antibody uptake into a human carcinoma xenograft model.. Br J Cancer.

[OCR_00735] Huston J. S., Levinson D., Mudgett-Hunter M., Tai M. S., Novotný J., Margolies M. N., Ridge R. J., Bruccoleri R. E., Haber E., Crea R. (1988). Protein engineering of antibody binding sites: recovery of specific activity in an anti-digoxin single-chain Fv analogue produced in Escherichia coli.. Proc Natl Acad Sci U S A.

[OCR_00743] Kiyokawa T., Williams D. P., Snider C. E., Strom T. B., Murphy J. R. (1991). Protein engineering of diphtheria-toxin-related interleukin-2 fusion toxins to increase cytotoxic potency for high-affinity IL-2-receptor-bearing target cells.. Protein Eng.

[OCR_00750] Laemmli U. K. (1970). Cleavage of structural proteins during the assembly of the head of bacteriophage T4.. Nature.

[OCR_00753] Landolfi N. F. (1991). A chimeric IL-2/Ig molecule possesses the functional activity of both proteins.. J Immunol.

[OCR_00756] LeBerthon B., Khawli L. A., Alauddin M., Miller G. K., Charak B. S., Mazumder A., Epstein A. L. (1991). Enhanced tumor uptake of macromolecules induced by a novel vasoactive interleukin 2 immunoconjugate.. Cancer Res.

[OCR_00763] Lei S. P., Lin H. C., Wang S. S., Callaway J., Wilcox G. (1987). Characterization of the Erwinia carotovora pelB gene and its product pectate lyase.. J Bacteriol.

[OCR_00770] Lorberboum-Galski H., FitzGerald D., Chaudhary V., Adhya S., Pastan I. (1988). Cytotoxic activity of an interleukin 2-Pseudomonas exotoxin chimeric protein produced in Escherichia coli.. Proc Natl Acad Sci U S A.

[OCR_00776] Lotze M. T., Chang A. E., Seipp C. A., Simpson C., Vetto J. T., Rosenberg S. A. (1986). High-dose recombinant interleukin 2 in the treatment of patients with disseminated cancer. Responses, treatment-related morbidity, and histologic findings.. JAMA.

[OCR_00782] Marks J. D., Hoogenboom H. R., Bonnert T. P., McCafferty J., Griffiths A. D., Winter G. (1991). By-passing immunization. Human antibodies from V-gene libraries displayed on phage.. J Mol Biol.

[OCR_00788] McCafferty J., Griffiths A. D., Winter G., Chiswell D. J. (1990). Phage antibodies: filamentous phage displaying antibody variable domains.. Nature.

[OCR_00793] Rosenberg S. A., Lotze M. T., Yang J. C., Aebersold P. M., Linehan W. M., Seipp C. A., White D. E. (1989). Experience with the use of high-dose interleukin-2 in the treatment of 652 cancer patients.. Ann Surg.

[OCR_00805] Smith K. A., Favata M. F., Oroszlan S. (1983). Production and characterization of monoclonal antibodies to human interleukin 2: strategy and tactics.. J Immunol.

[OCR_00810] Strauch K. L., Johnson K., Beckwith J. (1989). Characterization of degP, a gene required for proteolysis in the cell envelope and essential for growth of Escherichia coli at high temperature.. J Bacteriol.

[OCR_00816] Towbin H., Staehelin T., Gordon J. (1979). Electrophoretic transfer of proteins from polyacrylamide gels to nitrocellulose sheets: procedure and some applications.. Proc Natl Acad Sci U S A.

[OCR_00822] Ward E. S., Güssow D., Griffiths A. D., Jones P. T., Winter G. (1989). Binding activities of a repertoire of single immunoglobulin variable domains secreted from Escherichia coli.. Nature.

[OCR_00828] Williams D. P., Parker K., Bacha P., Bishai W., Borowski M., Genbauffe F., Strom T. B., Murphy J. R. (1987). Diphtheria toxin receptor binding domain substitution with interleukin-2: genetic construction and properties of a diphtheria toxin-related interleukin-2 fusion protein.. Protein Eng.

[OCR_00835] Yokota T., Milenic D. E., Whitlow M., Schlom J. (1992). Rapid tumor penetration of a single-chain Fv and comparison with other immunoglobulin forms.. Cancer Res.

